# Melatonin reduces bacterial translocation by preventing damage to the intestinal mucosa in an experimental severe acute pancreatitis rat model

**DOI:** 10.3892/etm.2013.1338

**Published:** 2013-10-10

**Authors:** XUECHENG SUN, YINGYING SHAO, YIN JIN, JIAPING HUAI, QIONG ZHOU, ZHIMING HUANG, JIANSHENG WU

**Affiliations:** Department of Gastroenterology and Hepatology, First Affiliated Hospital of Wenzhou Medical College, Wenzhou, Zhejiang 325000, P.R. China

**Keywords:** acute pancreatitis, bacterial translocation, melatonin, intestine barrier dysfunction, microvilli structure

## Abstract

Recent studies have demonstrated that melatonin significantly decreased all studied acute pancreatitis-associated inflammatory parameters, in addition to reducing apoptosis and necrosis associated with pancreatic injury. However, the effect of melatonin on gut barrier dysfunction and bacterial translocation has not been fully elucidated. This study aimed to investigate the protective effects of melatonin on intestinal integrity in a rat model of severe acute pancreatitis (SAP) to evaluate whether melatonin prevented intestine barrier dysfunction and reduced bacterial translocation. Forty male Sprague Dawley (SD) rats were randomly divided into three groups, with 8 rats in the sham operation (SO) group, 18 rats in the SAP group and 14 SAP rats in the melatonin treatment (MT) group. SAP was induced by retrograde injection of 4% taurocholate into the biliopancreatic duct. Melatonin was administered 30 min prior to taurocholate injection in the melatonin-treated rats. All rats were sacrificed 24 h subsequent to pancreatitis induction. Real-time fluorescence quantitative polymerase chain reaction was used to detect and quantify *Escherichia coli* (*E. coli*) O157 in postcava blood. The microvilli structure was also analyzed with transmission electron microscopy. The level of *E. coli* DNA in the MT group was significantly lower than in rats in the SAP group. No *E. coli* DNA was detected in the control group. Villus height and crypt depth in the ileum were significantly higher in the MT and control groups compared to the SAP group, and were significantly higher in the MT group than in the SAP group. These results suggested that melatonin prevented gut barrier dysfunction and reduced bacterial translocation, resulting in reduced pancreatic-associated infections and decreased early mortality rates.

## Introduction

It is well known that severe acute pancreatitis (SAP) is associated with high mortality rates. To date, no specific therapies have been developed. The annual global incidence of SAP is 20–100 per 100,000, resulting in mortality rates of 20–30%. The majority of the morbidity and mortality associated with SAP is the result of complications associated primarily with bacterial infections (1). The first peak in mortality occurs within the first week subsequent to the onset of symptoms and is characterized by systemic inflammatory response syndrome (SIRS) associated with multiple organ damage (2). The second peak is often observed 3–4 weeks following admission. The main cause associated with this late deterioration and systemic organ failure is bacterial superinfection of the pancreas resulting in sepsis (3–6). Bacterial translocation is considered the main cause of superinfection resulting in pancreatic necrosis that peaks during the first four days following the onset of symptoms (7). Subsequently, changes to intestinal motility and flora, mucosal barrier function and immune responses may lead to bacterial translocation, resulting in subsequent superinfection and pancreatic necrosis (8,9). The pathological mechanism and the route of bacterial translocation associated with severe acute necrotizing pancreatitis are not completely understood. Hematogenous or lymphatic dissemination of bacteria (10,11) have been suggested, and it is generally accepted that bacterial translocation from the gut is the primary cause of secondary pancreatic infections.

Diamine oxidase (DAO) is an effective biomarker that reflects the integrity and mucosal function of the small intestine. As a measure of small intestine barrier function, changes in the concentrations of DAO in the serum and the mucosa of the small intestine may be determined.

Melatonin is secreted by the pineal gland, although its main source has been observed to be the gastrointestinal tract (12). Melatonin is known to be important in the seasonal reproduction of certain species and in the regulation of circadian rhythms. The level of melatonin in the gastrointestinal tract is ~400-fold higher than the level of melatonin in the pineal gland, and the concentrations of melatonin in the gastrointestinal tract are 10–100-fold higher than in plasma. Melatonin also has anti-inflammatory and antioxidant properties that reduce ischemia/reperfusion injury and aid in immune defense. Previous studies have demonstrated that melatonin significantly decreased all studied acute pancreatitis (AP)-associated inflammatory parameters, in addition to reducing apoptosis and necrosis associated with pancreatic injury (13–17). Col *et al*(13) observed that intraperitoneal melatonin injections reduced the quantity of malonyldialdehyde (MDA), and increased the levels of superoxide dismutase (SOD) and glutathione (GSH), which are associated with oxidative stress in pancreatic tissue. Additional studies (14–17) have observed that melatonin reduced the occurrence and development of AP, suggesting that melatonin may ameliorate AP severity through its influence on cytokines, such as tumor necrosis factor (TNF)-α.

## Materials and methods

### Animals

Twenty-seven clean-grade, male Sprague Dawley (SD) rats weighing 200–250 g were purchased from the Experimental Animal Center of Wenzhou Medical College (Wenzhou, China). The animals were maintained under standard conditions of 12-h light/dark cycles in a temperature-controlled room with free access to standard rat pellets and water. All animals were maintained in the laboratory for one week and were deprived of food for 12 h prior to experimentation (rats had free access to water throughout the experimental period).

### Ethics statement

This experiment was approved by and performed in accordance with the guidelines for animal use of the Experimental Animal Center of Wenzhou Medical College.

### Animal groups and procedures

SD rats were randomly divided into the sham operation group (SO group, n=8), the SAP group (n=18) or the melatonin treatment group (MT group, n=14). SAP was induced through retrograde infusion of 4% taurocholate (1 ml/kg body weight; Sigma-Aldrich, St. Louis, MO, USA) into the biliopancreatic duct, following the clamping of the hepatic duct. In the SO group, the procedure was terminated subsequent to cannulating the biliopancreatic duct by penetrating the duodenum with a 24-gauge catheter. In the MT group, melatonin (50 mg/kg body weight; Sigma-Aldrich) was administered 30 min prior to the injection of taurocholate. Twenty-four hours subsequent to SAP induction, rats from each group were anesthetized with 10% chloral hydrate (30 mg/kg body weight), the abdomen was opened and the ileum tissues adjacent to the cecum were rapidly collected and divided in two. The first tissue sample from each rat was cut into several small pieces (1×1 mm), immediately fixed in 2.5% glutaraldehyde in phosphate-buffered saline (PBS, pH 7.2) and embedded in epoxy resin, prior to ultra-thin sections being prepared using conventional procedures. These sections were later examined with a JEM-1230 transmission electron microscope (JEOL Ltd., Tokyo, Japan). The second tissue sample was weighed and stored at −80°C in PBS for later anaylsis of DAO levels using enzyme-linked immunosorbent assay (ELISA). Blood samples (2 ml) from each rat in each group were collected via a postcava puncture and split in two. Half of the blood sample was allowed to clot for 20 min at room temperature and centrifuged at 3,000 × g for 20 min, prior to the serum being collected for the measurement of DAO levels using ELISA. The second portion was collected in germ-free microtubes with anticoagulants and stored at −20°C for the later quantification of *Escherichia coli* (*E. coli)* O157 using real-time-fluorescence quantitative polymerase chain reaction (RT-FQ-PCR). The rats were sacrificed by exsanguination at the end of the experiment.

### Transmission electron microscopy

The ileum tissues were cut into small pieces (1×1 mm) and immediately fixed in 2.5% glutaraldehyde in PBS and stored at 4°C overnight. The ileum segments were then washed in 0.1 M PBS three times (15 min each) and fixed in 1% osmium tetroxide for 1 h. One hour later, the segments were washed in 0.1 M PBS a further three times for 15 min each. The samples were then successively dehydrated in 50, 70, 80 and 90% acetone, respectively, for 15 min at each concentration, prior to being placed in 100% acetone twice for 10 min each. Subsequently, the segments were embedded in acetone and Epon resin (v/v=1:1), stored at 37°C for 2 h, embedded in acetone and Epon resin (v/v=1:4) and stored at 37°C overnight. The following day, the segments were embedded in Epon resin and stored at 45°C for 2 h. The segments were then cut longitudinally through the intestinal villi into 70–90 μm ultra-thin sections using a Reichert ultra-thin microtome (Reichert Inc., Buffalo, NY, USA). Subsequently, the ultra-thin sections were stained with uranyl acetate and lead citrate, and examined using a JEM-1230 transmission electron microscope.

### RT-FQ-PCR analysis

The postcava blood was removed from storage at −20°C and placed into liquid nitrogen. *E. coli* O157 DNA in the postcava blood was extracted using extraction solution (Da An Gene, Zhongshan, China) in accordance with the manufacturer’s instructions, and extraction was confirmed using agarose gel electrophoresis. *E. coli* O157 DNA was amplified using Taq DNA polymerase (Da An Gene). PCR was carried out using the following primers: Forward primer 5′-CAGATCCGGCAAGGTATTGT-3′ and reverse primer 5′-TGAGCGTTAAGCAGGTGATG-3′. The reporter dye 6-carboxyfluorescein (FAM) and the quencher dye Texas Red (Sulforhodamine 101) were conjugated at the 5′ and 3′ ends of the fluorescent probe, respectively. The reaction mixture (25 μl) consisted of 3 μl Taq DNA polymerase, 1 μl 2.5 mmol/l deoxyribonucleoside triphosphates (dNTPs), 5 μl DNA template, 1 μl each primer, 1 μl Taqman probe and 13 μl MgCl_2_, and the volume was adjusted with double-distilled water (ddH_2_O). Genomic DNA purified from *E. coli* was used as a positive control and ddH_2_O was used as a negative control. The following PCR cycling conditions were used: 50°C for 2 min, 95°C for 15 min, and 40 cycles at 94°C for 15 sec and 60°C for 45 sec. The RT-PCR results were recorded using a 7500 Sequence Detection system (Applied Biosystems Inc., Carlsbad, CA, USA).

### Serum and ileum mucosal DAO level measurements

The serum and ileum tissue samples were removed from storage at −80°C and placed into liquid nitrogen. When thawed, the samples were maintained at 2–8°C. The ileum tissues were homogenized by hand or using a tissue grinder, centrifuged at 3,000 rpm for 20 min and the supernatants were then collected to measure the DAO levels. Serum and ileum mucosal DAO assays were performed using a rat DAO ELISA kit (Daweike Biotechnology, Shanghai, China) according to the manufacturer’s instructions. Duplicate assays were performed on all DAO specimens. The DAO assay range was 700–800 U/l.

### Statistical analysis

Block randomization was used to assign animals into the three groups. Data are expressed as the arithmetic mean ± standard deviation (SD). Fisher probabilities in 2×2 tables were used to analyze the early mortality rates of the SD rats and the positive *E. coli* rates in the postcava blood. One-way analysis of variance (ANOVA) was used to investigate the differences among the three experimental groups, and comparisons were performed between samples from the same experiment and time-points to check for statistical significance. P≤0.05 was considered to indicate a statistically significant difference. Statistical analyses were performed using the SPSS software (version 17.0; SPSS Inc., Chicago, IL, USA).

## Results

### Mortality rates

Ten rats in the SAP group died 8–12 h subsequent to SAP induction (mortality rate of 55.56%). Three rats in the MT group died 12–24 h subsequent to SAP induction, resulting in a 21.43% mortality rate. The mortality rate in the MT group was significantly lower than that in the SAP group (P<0.05). No rats in the SO group died.

### Transmission electron microscopy

**Figure 1:**
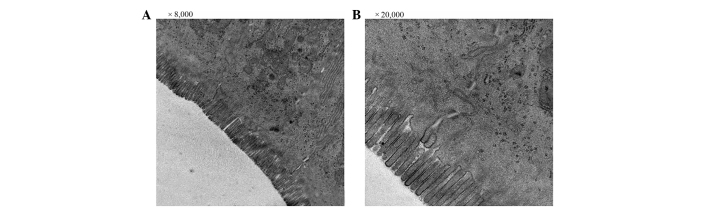
Images of the sham operation (SO) group obtained via transmission electron microscopy with uranyl acetate staining. (A) The intestinal villi and goblet epithelial cells from the SO group were intact (magnification, ×8,000). (B) The intestinal villi and goblet epithelial cells of the SO group were intact, and the mitochondria, endoplasmic reticulum, ribosomes and other cellular organelles were normal (magnification, ×20,000).

**Figure 2:**
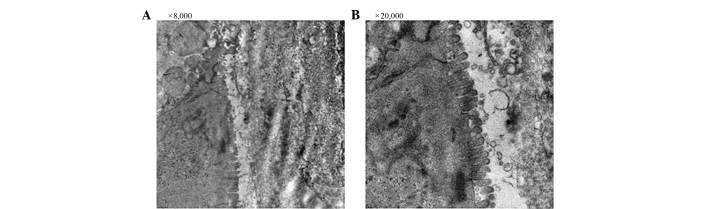
Images of the severe acute pancreatitis (SAP) group obtained via transmission electron microscopy with uranyl acetate staining. (A) The intestinal villi and goblet epithelial cells of the SAP group were diffuse and the intestinal villi were absent in various areas of the sample examined (magnification, ×8,000). (B) The intestinal villi and goblet epithelial cells of the SAP group were diffuse, and the intestinal villi were absent in various locations of the sample examined. Mitochondrial membranes and mitochondrial cristae were absent and had changed into vacuoles. The amount of rough endoplasmic reticulum was increased and enlarged and ribosome numbers were significantly increased (magnification, ×20,000).

**Figure 3:**
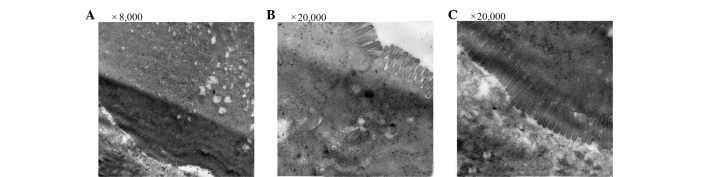
Images of the melatonin treatment (MT) group obtained via transmission electron microscopy with uranyl acetate staining. (A) The integrity of the intestinal villi and goblet epithelial cells in the MT group animals was maintained (magnification, ×8,000). (B) The intestinal villi and goblet epithelial cells of the MT group were intact (magnification, ×20,000). (C) Microrganelles, endoplasmic reticulum, ribosomes and other organelles remained intact in samples examined from rats in the MT group (magnification, ×20,000).

The integrity of the intestinal villi and goblet epithelial cells of the rats in the SO group was maintained throughout the course of the experiment (Fig. 1A and B). The mitochondria, endoplasmic reticulum, ribosomes and other cellular organelles were normal following examination at a magnification of ×20,000 (Fig. 1B). The intestinal villi and goblet epithelial cells of the SAP group presented with a loss of integrity, and the intestinal villi were absent throughout the sample examined (Fig. 2A and B). The mitochondrial membrane and cristae were absent and cristae had changed into vacuoles. The amount of rough endoplasmic reticulum was increased and enlarged, and the ribosomes were distinctly increased in size at a magnification of ×20,000 (Fig. 2B). The integrity of the intestinal villi and goblet epithelial cells of rats in the MT group was maintained (Fig. 3A and B). The mitochondria, endoplasmic reticulum, ribosomes and other organelles were unchanged (Fig. 3C).

### Measurements of intestinal integrity

**Figure 4:**
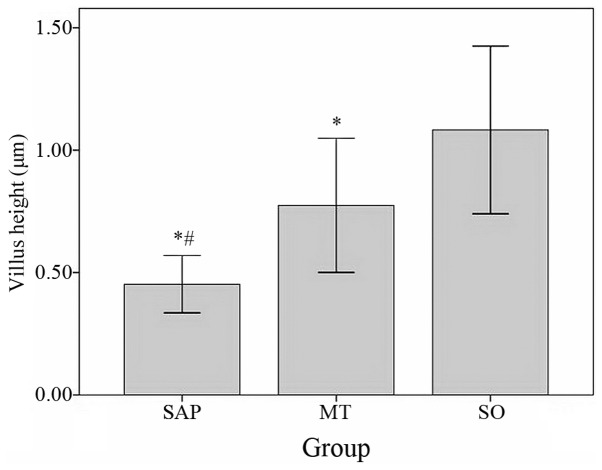
Villus height of the ileum mucosa (μm). ^*^P≤0.05 vs. SO group; ^#^P≤0.05 vs. MT group. Data are expressed as the mean ± standard deviation. SAP, severe acute pancreatitis; MT, melatonin treatment; SO, sham operation.

Compared with the villus height in the SO group (1.08250±0.171193 μm), the villus height in the SAP group (0.45250±0.058493 μm, P<0.05) and the MT group (0.77455±0.137067 μm, P<0.05) were significantly lower. The villus height in the MT group was significantly higher than in the SAP group (P<0.05; Fig. 4).

**Figure 5:**
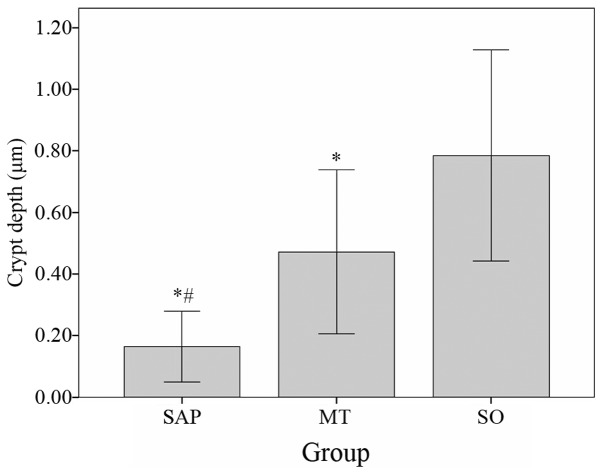
Crypt depth of the ileum mucosa (μm). ^*^P≤0.05 vs. SO group; ^#^P≤0.05 vs. MT group. Data are expressed as the mean ± standard deviation. SAP, severe acute pancreatitis; MT, melatonin treatment; SO, sham operation.

The crypt depth in the SO group (0.78500±0.171548 μm) was significantly increased compared with that in the SAP group (0.16500±0.057570 μm, P<0.05) and the MT group (0.47182±0.133178 μm, P<0.05). The crypt depth of the MT group was significantly deeper than in the SAP group (Fig. 5).

### E. coli O157 quantification in postcava blood

**Figure 6:**
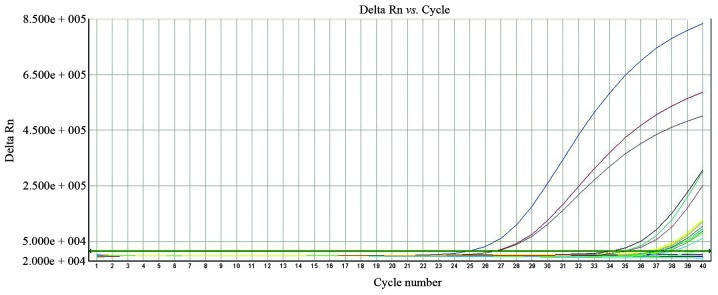
*E. coli* O157 polymerase chain reaction (PCR) augmentation dynamics curve.

The concentration of *E. coli* DNA (Ct value) in the postcava blood in the MT group was significantly lower than the levels in the rats in the SAP group. No *E. coli* DNA was detected in the animals in the SO group (Table I; Fig. 6). *E. coli* DNA in postcava blood was significantly lower in the MT group compared to the SAP group (Table I).

### Serum and ileum mucosal DAO level

**Figure 7:**
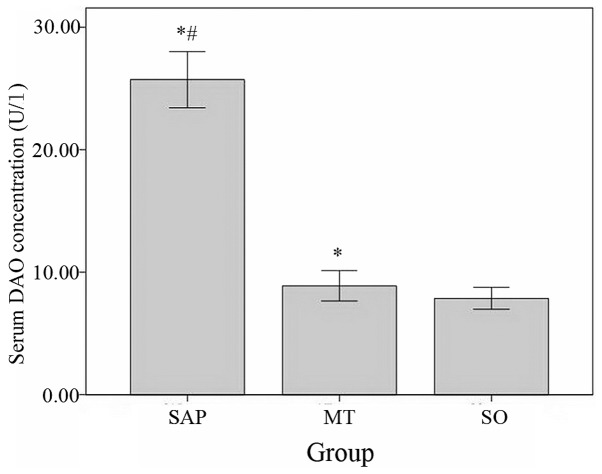
Serum diamine oxidase (DAO) concentrations (U/l). ^*^P>0.05 vs. SO group; ^#^P≤0.05 vs. MT group. Data are expressed as the mean ± standard deviation. SAP, severe acute pancreatitis; MT, melatonin treatment; SO, sham operation.

The levels of DAO in the serum were significantly higher in the SAP group compared with the levels observed in the MT group and significantly higher in the MT group than in the control group (Fig. 7).

**Figure 8:**
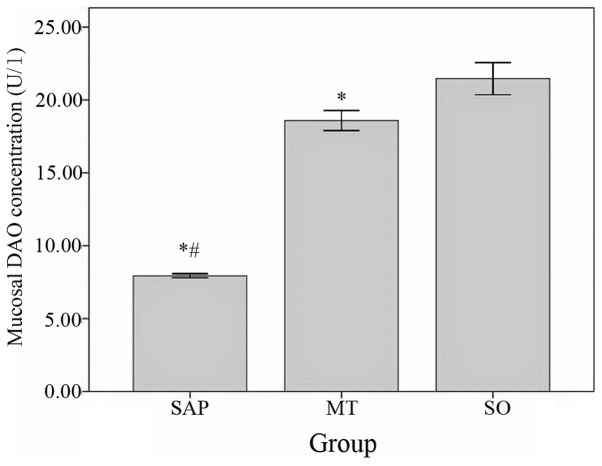
Mucosal diamine oxidase (DAO) concentrations (U/l). ^*^P>0.05 vs. SO group; ^#^P≤0.05 vs. MT group. Data are expressed as the mean ± standard deviation. SAP, severe acute pancreatitis; MT, melatonin treatment; SO, sham operation.

The levels of DAO in the ileum mucosa were significantly lower in the SAP group than in the MT group and significantly lower in the MT group than in the control group (Fig. 8).

## Discussion

Lichtman (18) suggested that the clinical outcome of patients presenting with SAP was significantly associated with bacteria crossing the intestinal barrier and then invading organ systems, resulting in superinfections associated with pancreatic necrosis. At present, bacterial translocation is considered to be the main cause of pancreatic superinfection and fatal sepsis (8,19). Ammori *et al*(3) reported that gut barrier function was disordered and that endotoxemia was associated with SAP. Furthermore, Cicalese *et al*(9) observed that fluorescent microspheres administered orally to rats prior to the induction of AP were able to be detected later in different organ systems, including the pancreas, liver or spleen. These experiments, as wells as other studies (20,21), demonstrated that the gut was the main source of bacterial translocation in AP. The aim of the present study was to evaluate whether melatonin was able to reduce bacterial translocation and ameliorate gut barrier dysfunction, resulting in an improved clinical course associated with reduced early mortality rates, in rats with SAP. It was therefore necessary to detect systemic bacterial dissemination early, prior to the development of systemic infections. Early dissemination was monitored by testing blood samples that were frequently positive in rats with pancreatitis, indicative of hematogenous dissemination. Several hypotheses regarding the spread of intestinal bacteria have been proposed. Although certain authors have suggested a lymphatic spread of enteric bacteria, others have suggested that bacteria cross the intestinal barrier and invade blood vessels (hematogenous dissemination). Furthermore, transductal infections via the biliary tract (either ascending or descending) and transperitoneal pathways have been proposed (10,21). In the present study, it was observed that melatonin treatment significantly reduced infection of postcava blood, prevented gut barrier dysfunction and subsequently reduced pancreatic superinfections. It was concluded that bacterial translocation occurred via mesenteric lymph nodes and subsequent hematogenous dissemination. This was in accordance with previous studies, which showed that bacterial translocation does not occur via transperitoneal pathways, but most likely via lymphatic spread (22,23) followed by hematogenous dissemination. Lichtman (18) suggested that bacterial cell wall components (such as lipopolysaccharide and peptidoglycan polysaccharide) allowed enteric bacteria to cross the intestinal barrier into the mesenteric lymph nodes, resulting in the subsequent spread of the bacteria throughout the body causing sepsis and multiple organ failure (MOF).

It is known that the small bowel has an important pathophysiological role in the infection process associated with pancreatic necrosis. Samel *et al*(20) observed that fluorescent bacteria translocated from the small bowel lumen into the pancreas. Fritz *et al*(24) suggested that bacterial translocation occurred through the small bowel rather than through the colon. A subsequent increase in intestinal permeability facilitated bacterial translocation (25), resulting in apoptosis of intestinal epithelial cells and/or alterations to tight junction integrity (26). Although the pathogenesis of intestinal bacterial translocation associated with SAP has yet to be elucidated, several mechanisms have been proposed, including: i) Altered permeability of the intestinal mucosa, ii) a disruption of the indigenous gut flora, and iii) decreased host defenses. SAP may be closely associated with these as well as other factors that may promote bacterial translocation. Widdison *et a1*(27) described a reduced clearance of *E. coli* from the circulation during SAP associated with impaired phagocytic and reticuloendothelial function. Evidence of bacterial translocation associated with enterogenic infections resulting in AP and multiple organ dysfunction syndrome (MODS) has led to a shift in focus onto the area of intestinal mucosal barrier integrity as a key player in preventing SAP.

The intestinal mucosal barrier is able to prevent the transport of harmful substances, including dangerous bacteria and/or toxins, from penetrating the intestinal wall, and maintains the stability of the internal environment (28,29). SAP, as well as surgery, trauma, chemotherapy, radiotherapy or severe infection, may damage the integrity and function of the intestinal mucosa. In the present study, transmission electron microscopy demonstrated that microvilli of the intestinal mucosa had reduced widths and heights, tight junctions were damaged and DAO levels were increased in SAP rats. Each of these parameters are capable of leading to increases in intestinal permeability (30–33), causing activation of endothelial cells, translocation of enteric bacteria and endotoxins, and the release of cytokines and inflammatory mediators that may result in the onset of SIRS and MODS.

DAO is a high-activity intracellular enzyme that metabolizes and catalyzes histamine, cadaverine and putrescine, and is predominantly present within the intestinal mucosa, placenta and kidney. However, it is also present in low levels in the plasma (34). DAO oxidizes putrescine into amino butyraldehyde and cyclized into pyrrole. The activity of DAO is closely correlated with intestinal villi height and protein synthesis. When the intestinal barrier is injured, intestinal mucosal cells exfoliate into the gut lumen, decreasing the activity of mucosal DAO. When DAO enters the lymphatic vessels and the blood stream, the plasma DAO levels are increased. Therefore, high plasma and low mucosal concentrations of DAO reflect impairment of the intestinal tract function. It has been suggested that the combined characterization of the ratio of urine lactulose to excretion and plasma DAO levels may be used as measures of intestinal mucosal function and integrity. The plasma DAO concentrations reflect the intestinal permeability more effectively and rapidly (35).

In the present study, alterations to ileal mucosal and serum DAO levels were characterized in order to evaluate the function of the small intestinal barrier and the permeability function in rats with SAP. The level of ileum mucosal DAO was decreased and the level of serum DAO was increased in the SAP group. These results indicated that damage to the intestinal barrier resulted in increased intestinal permeability that occurred during the early stages of SAP. These observations were confirmed by the level of *E. coli* O157 (Ct value) detected in postcava blood by RT-FQ-PCR in the SAP group.

In 1991, Lanas *et al* first proposed that melatonin was an antioxidant, and it was subsequently tested in a number of toxicity studies (36–38). Melatonin readily protected the gastric and enteric mucosa from damage caused by various factors, including ischemia/reperfusion (39), stress (40) and ethanol (41). The present results suggested that melatonin prevented (or reduced) the severity of experimental AP by increasing antioxidant enzyme activity. To date, few studies have examined the effects of melatonin on gut barrier dysfunction and intestinal bacterial translocation, which is an important trigger that drives the development of SIRS and MODS. The main observations of this study were that melatonin reduced intestinal bacterial translocation and reduced pancreatic infection and early mortality rates by protecting the function and structure of the intestinal mucosa.

The results of the present study indicated that melatonin protected the small intestinal villi from damage caused by taurocholate-induced SAP, subsequently preventing intestinal barrier dysfunction and significantly reducing intestinal permeability, which, in turn, prevented intestinal bacterial translocation. The villus height in the MT group was significantly higher than in the SAP group and the crypt depth of the MT group was significantly deeper than in the SAP group. The level of ileum mucosal DAO was decreased and the level of serum DAO was increased in rats not treated with melatonin (SAP group). However, alterations in the ileum mucosal and serum DAO levels were not different in the MT group compared with rats in the SO group. The level of *E. coli* O157 also differed between groups. This study demonstrated that melatonin treatment of rats presenting with SAP protected intestinal mucosal cells against mechanical and chemical damage, attenuated damage to the small intestines, reduced intestinal bacterial translocation and reduced early mortality rates. Thus, melatonin may reduce intestinal bacterial translocation by alleviating intestinal injury.

In conclusion, the results of the study demonstrated that intestinal bacterial translocation may be associated with damage to the intestinal mucosal barrier. Melatonin is potentially capable of reducing intestinal bacterial translocation by preventing damage to the intestinal mucosa.

## Figures and Tables

**Table I:** *E. coli* DNA in postcava blood.

Groups	N	*E. coli* DNA (n)	Positive rate (%)	Ct-value
SAP	8	0	0.00	0
MT	11	1	9.09	35.1081±3.2873
SO	8	5	62.50^a^	29.4466±4.74451^b^

a P≤0.05 vs. MT group;

b P>0.05 vs. MT group.

Measurement data are expressed as the mean ± standard deviation. SAP, severe acute pancreatitis; MT, melatonin treatment; SO, sham operation; N, number.

## References

[1] Petrov MS, Shanbhag S, Chakraborty M, Phillips AR, Windsor JA (2010). Organ failure and infection of pancreatic necrosis as determinants of mortality in patients with acute pancreatitis. Gastroenterology.

[2] Bettinger JR, Grendell JH (1991). Intracellular events in the pathogenesis of acute pancreatitis. Pancreas.

[3] Ammori BJ, Fitzgerald P, Hawkey P, McMahon MJ (2003). The early increase in intestinal permeability and systemic endotoxin exposure in patients with severe acute pancreatitis is not associated with systemic bacterial translocation: molecular investigation of microbial DNA in the blood. Pancreas.

[4] Gregoric P, Sijacki A, Stankovic S (2010). SIRS score on admission and initial concentration of IL-6 as severe acute pancreatitis outcome predictors. Hepatogastroenterology.

[5] Slavin J, Neoptolemos JP (2001). Antibiotic prophylaxis in severe acute pancreatitis - what are the facts?. Langenbecks Arch Surg.

[6] Gloor B, Müller CA, Worni M, Martignoni ME, Uhl W, Büchler MW (2001). Late mortality in patients with severe acute pancreatitis. Br J Surg.

[7] Tarpila E, Nyström PO, Franzén L, Ihse I (1993). Bacterial translocation during acute pancreatitis in rats. Eur J Surg.

[8] van Minnen LP, Blom M, Timmerman HM, Visser MR, Gooszen HG, Akkermans LM (2007). The use of animal models to study bacterial translocation during acute pancreatitis. J Gastrointest Surg.

[9] Cicalese L, Sahai A, Sileri P (2001). Acute pancreatitis and bacterial translocation. Dig Dis Sci.

[10] Runkel NS, Rodriguez LF, Moody FG (1995). Mechanisms of sepsis in acute pancreatitis in opossums. Am J Surg.

[11] de las Heras G, Forcelledo JL, Gutiérrez JM (2000). Selective intestinal bacterial decontamination in experimental acute pancreatitis. Gastroenterol Hepatol.

[12] Bubenik GA, Hacker RR, Brown GM, Bartos L (1999). Melatonin concentrations in the luminal fluid, mucosa, and muscularis of the bovine and porcine gastrointestinal tract. J Pineal Res.

[13] Col C, Dinler K, Hasdemir O, Buyukasik O, Bugdayci G (2010). Oxidative stress and lipid peroxidation products: effect of pinealectomy or exogenous melatonin injections on biomarkers of tissue damage during acute pancreatitis. Hepatobiliary Pancreat Dis Int.

[14] Chen HM, Hsu JT, Chen JC, Ng CJ, Chiu DF, Chen MF (2005). Delayed neutrophil apoptosis attenuated by melatonin in human acute pancreatitis. Pancreas.

[15] Jaworek J, Bonio J, Leja-Szpa A (2002). Sensory nerves in central and peripheral control of pancreatic integrity by leptin and melatonin. J Physiol Pharmacol.

[16] Jaworek J, Leja-Szpak A, Bonior J (2003). Protective effect of melatonin and its precursor L-tryptophan on acute pancreatitis induced by caerulein overstimulation or ischemia/reperfusion. J Pineal Res.

[17] Gülben K, Ozdemir H, Berberoğlu U (2010). Melatonin modulates the severity of taurocholate-induced acute pancreatitis in the rat. Dig Dis Sci.

[18] Lichtman SM (2001). Bacterial [correction of baterial] translocation in humans. J Pediatr Gastroenterol Nutr.

[19] Arendt T, Wendt M, Olszewski M, Falkenhagen U, Stoffregen C, Fölsch UR (1997). Cerulein-induced acute pancreatitis in rats - does bacterial translocation occur via a transperitoneal pathway?. Pancreas.

[20] Samel S, Lanig S, Lux A (2002). The gut origin of bacterial pancreatic infection during acute experimental pancreatitis in rats. Pancreatology.

[21] Yasuda T, Takeyama Y, Ueda T (2006). Breakdown of intestinal mucosa via accelerated apoptosis increases intestinal permeability in experimental severe acute pancreatitis. J Surg Res.

[22] Wazna E, Górski A (2005). Bacterial translocation and its clinical significance. Postepy Hig Med Dosw (Online).

[23] Marotta F, Geng TC, Wu CC, Barbi G (1996). Bacterial translocation in the course of acute pancreatitis: beneficial role of nonabsorbable antibiotics and lactitol enemas. Digestion.

[24] Fritz S, Hackert T, Hartwig W (2010). Bacterial translocation and infected pancreatic necrosis in acute necrotizing pancreatitis derives from small bowel rather than from colon. Am J Surg.

[25] Wang X, Andersson R, Soltesz V, Leveau P, Ihse I (1996). Gut origin sepsis, macrophage function, and oxygen extraction associated with acute pancreatitis in the rat. World J Surg.

[26] Hać S, Dobosz M, Kaczor JJ (2005). Neutrophil engagement and septic challenge in acute experimental pancreatitis in rats. World J Gastroenterol.

[27] Widdison AL, Karanjia ND, Reber HA (1994). Routes of spread of pathogens into the pancreas in a feline model of acute pancreatitis. Gut.

[28] Garside P, Millington O, Smith KM (2004). The anatomy of mucosal immune responses. Ann N Y Acad Sci.

[29] Kiyono H, Kweon MN, Hiroi T, Takahashi I (2001). The mucosal immune system: from specialized immune defense to inflammation and allergy. Acta Odontol Scand.

[30] Meriläinen S, Mäkelä J, Koivukangas V (2012). Intestinal bacterial translocation and tight junction structure in acute porcine pancreatitis. Hepatogastroenterology.

[31] Besselink MG, van Santvoort HC, Renooij W (2009). Intestinal barrier dysfunction in a randomized trial of a specific probiotic composition in acute pancreatitis. Ann Surg.

[32] Takahashi Y, Fukushima J, Fukusato T (2005). Prevalence of ischemic enterocolitis in patients with acute pancreatitis. J Gastroenterol.

[33] Penalva JC, Martínez J, Laveda R (2004). A study of intestinal permeability in relation to the inflammatory response and plasma endocab IgM levels in patients with acute pancreatitis. J Clin Gastroenterol.

[34] Takagi K, Nakao M, Ogura Y, Nabeshima T, Kunii A (1994). Sensitive colorimetric assay of serum diamine oxidase. Clin Chim Acta.

[35] Luan ZG, Zhang H, Ma XC, Zhang C, Guo RX (2010). Role of high-mobility group box 1 protein in the pathogenesis of intestinal barrier injury in rats with severe acute pancreatitis. Pancreas.

[36] Galano A, Tan DX, Reiter RJ (2011). Melatonin as a natural ally against oxidative stress: a physicochemical examination. J Pineal Res.

[37] Hardeland R, Tan DX, Reiter RJ (2009). Kynuramines, metabolites of melatonin and other indoles: the resurrection of an almost forgotten class of biogenic amines. J Pineal Res.

[38] Peyrot F, Ducrocq C (2008). Potential role of tryptophan derivatives in stress responses characterized by the generation of reactive oxygen and nitrogen species. J Pineal Res.

[39] Cuzzocrea S, Costantino G, Mazzon E, Micali A, De Sarro A, Caputi AP (2000). Beneficial effects of melatonin in a rat model of splanchnic artery occlusion and reperfusion. J Pineal Res.

[40] Kato K, Murai I, Asai S (1997). Central effect of melatonin against stress-induced gastric ulcers in rats. Neuroreport.

[41] Melchiorri D, Sewerynek E, Reiter RJ, Ortiz GG, Poeggeler B, Nisticò G (1997). Suppressive effect of melatonin administration on ethanol-induced gastroduodenal injury in rats in vivo. Br J Pharmacol.

